# Editorial: Europeans and xenotransplantation

**DOI:** 10.3389/ti.2026.17780

**Published:** 2026-10-07

**Authors:** Emanuele Cozzi, David K. C. Cooper

**Affiliations:** 1 Department of Cardiac, Thoracic, Vascular Sciences and Public Health, University of Padova Medical School, Padova, Italy; 2 Thomas E. Starzl Transplantation Institute, University of Pittsburgh Medical Center, Pittsburgh, PA, United States

**Keywords:** advancements, cellular xenografts, contribution of Europeans, organ xenografts, xenotransplantation science

Recent developments in the field of xenotransplantation have attracted considerable attention and constitute a major turning point for this scientific discipline. These recent investigations illustrate the major progress that has taken place in the last decade. In particular, advances in genetic-engineering techniques have allowed the generation of donor pigs whose organs are increasingly compatible with humans from both an immunological and physiological perspective. In addition, the use of novel immunosuppressants has contributed to major advances in preclinical studies in which life-supporting pig organs have been able to sustain the lives of many nonhuman primates for more than a year, and even for 5 years in one case.

Such impressive results have opened the way for the first xenotransplantation procedures in human patients. The initial reports on the clinical results obtained to date may be considered by many to be perhaps mixed but they provide considerable encouragement. They have drawn attention to several aspects that require further investigation and, although the road remains an uphill climb, we are encouraged that further progress will likely be made in the near future. Indeed, as never before, xenotransplantation is now perceived as a probable solution to the world-wide shortage of human organs.

When we were invited by the Editor-in-Chief of *Transplant International,* to act as Guest Editors for a special issue of the journal on the latest developments in the field of xenotransplantation, we realized that many review articles had been published during the previous 24 months on various aspects of xenotransplantation. How could we throw some new light on the topic or address it from a novel perspective? As *Transplant International* is the official journal of the European Society for Organ Transplantation, we decided to concentrate attention on research carried out largely by authors who were born or educated or are currently living in Europe. In addition, we decided to invite a handful of clinical experts at the forefront of allotransplantation in Europe (with or without a special interest in xenotransplantation) to share with us their opinions on the current status of xenotransplantation and how they anticipate it will be introduced clinically.

Although much of the research into xenotransplantation has been carried out at centres in the USA, in many cases the work has actually involved a large number of Europeans who had either relocated permanently or temporarily to the USA. We therefore began by summarizing the many contributions of Europeans to (i) pig organ xenotransplantation (Iwanczyk et al.) and (ii) pig islet and cell xenotransplantation (Bottino et al.). Much of this work was carried out by young surgeons or physicians gaining experience in research before returning to their home countries in Europe, where several are now leaders in the field of transplantation.

Of those who remained in the USA, islet xenotransplantation, in particular, has benefitted by the sustained efforts of Europeans such as Bernhard Hering (Germany) and Rita Bottino (Italy). More recently, Pierre Gianello and his colleague, Nizar Mourad, in Belgium, working with Cesare Galli (Italy), have introduced a novel genetic modification by which pig pancreatic islets may produce much more insulin than those from wild-type pigs (Mourad and Gianello). As pigs need and produce less insulin than humans, these specific gene-edits may enable normoglycemia to be achieved in a diabetic recipient by the transplantation of fewer pig islets.

There have been several contributions by Europeans on both sides of the Atlantic in such areas as (i) the genetic engineering of the organ-source pigs, e.g., by the late David White (UK), Eckhard Wolf (Germany), and Cesare Galli (Galli; Ali et al.), (ii) the development of an effective immunosuppressive regimen, e.g., by Leo Buhler (Switzerland), who demonstrated the efficacy of CD40/CD154 co-stimulation blockade in xenotransplantation (Sanatkar et al.), (iii) pig liver xenotransplantation, e.g., Burcin Ekser (Turkey and Italy) (Sucu et al.), and (iv) the potential problems of infectious complications in xenotransplantation, in which several Europeans have become acknowledged leaders in the field, e.g., Joachim Denner (Germany), Linda Scobie (UK), and Nicolas Mueller (Switzerland) (Mueller and Scobie; Denner). In the important realm of basic science research, Joerg Seebach (Switzerland) (Galdina et al.) and Robert Rieben (Switzerland) (Gultom and Rieben) have both contributed significantly over many years.

Several Europeans (particularly from Erasmus University in The Netherlands) contributed to progress in pig heart xenotransplantation, including an assessment of the first α1,3-galactosyltransferase gene-knockout (GTKO) pigs (Iwanczyk et al.). Subsequently, the Munich group, founded and led by Bruno Reichart (Germany), played a leading role in establishing successful orthotopic pig heart transplantation in nonhuman primates, which had been elusive until then (Längin et al.). Progress in pig kidney and lung xenotransplantation has also progressed through the work of Europeans in the USA and in Europe (Iwanczyk et al.; Le Bas-Bernardet et al.; Takemoto et al.). The importance of the pig-to-nonhuman primate model, in which most of the advances we have seen over the past 40 years were developed, has been emphasized, particularly when compared with studies in human decedents (Cooper et al.). Commentaries on pig organ xenotransplantation have been contributed to this collection of papers by leaders in the field of organ allotransplantation (Karadagi and Oniscu; Böhmig et al.; Piemonti et al.). Finally, this special issue also includes a paper illustrating aspects of regulation of xenotransplantation in Europe (Tönjes).

We hope you will find the collected papers informative with regard to the important, and often overlooked, role that Europeans have played in the research that has enabled clinical trials of xenotransplantation to be initiated.

